# Alanyl-Glutamine Protects Mice against Methionine- and Choline-Deficient-Diet-Induced Steatohepatitis and Fibrosis by Modulating Oxidative Stress and Inflammation

**DOI:** 10.3390/nu14183796

**Published:** 2022-09-15

**Authors:** Jiaji Hu, Yigang Zheng, Hanglu Ying, Huabin Ma, Long Li, Yufen Zhao

**Affiliations:** 1 Institute of Drug Discovery Technology, Ningbo University, Ningbo 315211, China; 2 Ningbo Institute of Marine Medicine, Peking University, Ningbo 315010, China; 3 State Key Laboratory of Chemical Oncogenomics, Tsinghua Shenzhen International Graduate School, Shenzhen 518055, China

**Keywords:** alanyl-glutamine, nonalcoholic steatohepatitis, oxidative stress, inflammation, fibrosis

## Abstract

Nonalcoholic steatohepatitis (NASH) is a common chronic liver disease with increasing prevalence rates over years and is associated with hepatic lipid accumulation, liver injury, oxidative stress, hepatic inflammation, and liver fibrosis and lack of approved pharmacological therapy. Alanyl-glutamine (Ala-Gln) is a recognized gut-trophic nutrient that has multiple pharmacological effects in the prevention of inflammation- and oxidative-stress-associated diseases. Nevertheless, whether Ala-Gln has a protective effect on NASH still lacks evidence. The aim of this study is to explore the influence of Ala-Gln on NASH and its underlying mechanisms. Here, C57BL/6 mice were fed a methionine- and choline-deficient (MCD) diet to establish the model of NASH, and Ala-Gln at doses of 500 and 1500 mg/kg were intraperitoneally administered to mice along with a MCD diet. The results showed that Ala-Gln treatment significantly attenuated MCD-induced hepatic pathological changes, lowered NAFLD activity score, and reduced plasma alanine transaminase (ALT), aspartate transaminase (AST) and lactate dehydrogenase (LDH) levels. Ala-Gln dramatically alleviated lipid accumulation in liver through modulating the expression levels of fatty acid translocase (FAT/CD36) and farnesoid X receptor (FXR). In addition, Ala-Gln exerted an anti-oxidant effect by elevating the activities of superoxide dismutase (SOD) and glutathione peroxidase (GPX). Moreover, Ala-Gln exhibited an anti-inflammatory effect via decreasing the accumulation of activated macrophages and suppressing the production of proinflammatory mediators. Notably, Ala-Gln suppressed the development of liver fibrosis in MCD-diet-fed mice, which may be due to the inhibition of hepatic stellate cells activation. In conclusion, these findings revealed that Ala-Gln prevents the progression of NASH through the modulation of oxidative stress and inflammation and provided the proof that Ala-Gln might be an effective pharmacological agent to treat NASH.

## 1. Introduction

Nonalcoholic fatty liver disease (NAFLD) is one of the most common chronic liver diseases with a prevalence of up to 20–30% worldwide, which has become a serious public health problem [1]. NAFLD consists of a wide range of liver disfunctions from isolated hepatic steatosis to nonalcoholic steatohepatitis (NASH) and other associated liver disorders [2]. NASH is a subtype of NAFLD, which is characterized with hepatocyte ballooning, inflammation, oxidative stress, and various degrees of fibrosis in liver and could progress to cirrhosis and even hepatocellular carcinoma [3]. Multiple damaging signals are involved in progression of NASH, including proinflammatory cytokines, reactive oxygen species, and fatty acids. However, there are still no approved medicines for the prevention of NASH pathophysiology in clinic. Therefore, novel pharmacological agents are urgently needed for the treatment of NASH [4].

L-glutamine is a conditionally essential amino acid in the human body, which can be acquired from a variety of foods including grains, dairy products, fish, and poultry [5]. In fact, it has been shown that L-glutamine plays a major role in various physiological processes, including nitrogen transport, intracellular redox homeostasis, and glucose and glutathione synthesis [6]. However, its lower solubility and stability limit the application of L-glutamine in clinical studies [7,8]. Alanyl-glutamine (Ala-Gln) is a dipeptide containing a glutamine and an alanine, which is a more stable and soluble form to effectively increase the availability of glutamine to the body, and has been frequently used as a nutritional supplement in critical patients [9,10]. Interestingly, as a glutamine-containing peptide, Ala-Gln is also a food-derived peptide mainly soured from Ala-Gln-containing peptides or proteins in a number of foods [11]. It has been observed that Ala-Gln administration can suppress inflammation and oxidative stress in various tissues [12,13,14]. Pretreatment with Ala-Gln in rats has been observed to have protective effect against hepatic ischemia-reperfusion injury [15]. Our previous study revealed the hepatoprotective function of Ala-Gln in endotoxin-triggered acute liver damage [16]. However, whether Ala-Gln prevents NASH and its underlying mechanism is still unclear.

In this research, we aimed to explore how Ala-Gln affects the progression of NASH. Here, we revealed the protective role of Ala-Gln in NASH by regulating oxidative stress and inflammation, which suggests that Ala-Gln is a promising pharmacological agent for NASH.

## 2. Materials and Methods

### 2.1. Animal Experiments

C57BL/6 male mice, 6–8 weeks old, were acquired from Vital River Laboratory Animal Tech Co., Ltd. (Beijing, China). All the mice were housed under thermostatic condition (21–23 °C) with a 12 h light–dark cycle with free access to food and water. To establish a NASH model, the model group with or without Ala-Gln treatment was fed with a methionine- and choline-deficient (MCD) diet, while the normal control group with or without Ala-Gln treatment was fed with a standard diet. The Ala-Gln control group was intraperitoneally injected with Ala-Gln (1500 mg/kg) each day. The low-dose Ala-Gln group was treated with MCD diet and Ala-Gln (500 mg/kg, i.p.) each day. The high-dose Ala-Gln group was treated with MCD diet and Ala-Gln (1500 mg/kg, i.p.) each day. All the animal studies were performed according to the approved guidelines of the animal ethics and welfare Committee of Ningbo University (No. AEWC-2020-5, approved on 15 May 2020).

### 2.2. Materials

Ala-Gln was synthesized and provided by Xiamen university as previously described [17]. Oil Red O (ORO) was purchased from Merck (Shanghai, China). The assay kits for hematoxylin–eosin (HE) staining and Masson’s Trichrome Staining were obtained from Solarbio Science & Technology Co., Ltd. (Beijing, China). MCD diet was acquired from Trophic Animal Feed High-Tech Co., Ltd. (Nantong, China).

### 2.3. Histology

Liver tissue samples were fixed with 4% paraformaldehyde (PFA) at 4 °C for 24 h, paraffinized, and then cut into sections (5 μm), which were stained with HE to assess liver morphological characteristics, and the NAFLD activity score was quantitated by a comprehensive evaluation of histopathological changes including steatosis, hepatocyte ballooning, inflammation, and fibrosis, as reported previously [18]. Masson trichrome staining was carried out to detect hepatic fibrosis. Lipid accumulation in liver was determined by ORO staining of frozen sections from 4% PFA-fixed liver tissues. Masson-positive pixels were quantified by ImageJ, while Oil-Red-O-positive pixels were measured by Image-pro plus.

### 2.4. IHC Assay

After they were deparaffinized and rehydrated, paraffin-embedded liver sections were heated in sodium citrate solution for antigen retrieval and then immersed in 3% hydrogen peroxide. The blocking was performed with goat serum, followed by incubation with anti-F4/80 antibody or anti-α-SMA antibody overnight at 4 °C. Thereafter, secondary antibodies were applied to the sections after washing at room temperature (RT) for 1 h incubation. After washing, the sections were visualized with 3,3-diaminobenzidine (DAB) and co-stained with hematoxylin. Quantification of F4/80- and α-SMA-positive pixels was performed using ImageJ software after imaging using a light microscope (Leica Microsystems, Shanghai, China).

### 2.5. Biochemical Assay

Plasma alanine transaminase (ALT), aspartate transaminase (AST), and lactate dehydrogenase (LDH) levels were analyzed using commercially available kits from Nanjing Jiancheng Bioengineering Institute (Nanjing, China) to assess the degree of liver injury. Hepatic malondialdehyde (MDA), superoxide dismutase (SOD) and glutathione peroxidase (GPX) activities were detected by biochemical assay kits (Nanjing Jiancheng Bioengineering Institute, Nanjing, China) to evaluate the degree of oxidative stress. Total triglycerides (TG) levels in the liver were measured by TG assay kit (BioVision, Milpitas, CA, USA) to estimate the accumulation of liver fat.

### 2.6. ELISA

The levels of TNF-α, IL-1β, RANTES, and MCP-1 in mouse peripheral plasma were detected using Elisa assay kits (Multi Sciences, Hangzhou, China) following the manufacturer’s protocols.

### 2.7. Western Blots

Proteins form liver tissues were extracted by lysis buffer from Beyotime Biotechnology (Shanghai, China) with protease inhibitors from Roche (Shanghai, China). After quantification by BCA assay kit (TransGen Biotech, Beijing, China), protein samples were separated on SDS-PAGE and transferred onto PVDF membranes (Merck Millipore, Shanghai, China). Membranes were blocked with 5% skimmed milk at RT for 1 h and then incubated over night at 4 °C with anti-α-SMA antibody (1:1000) and anti-GAPDH antibody (1:10,000). Next, all membranes were washed and then incubated with HRP-conjugated goat anti-rabbit or goat anti-mouse antibody for 1 h at RT. Finally, protein levels were visualized using ChemiDoc MP Image System (Bio-Rad, Shanghai, China).

### 2.8. Real-Time PCR

RNA extraction kit from Tiangen Biotech Co., Ltd. (Beijing, China) was used to isolate RNA from liver tissues in accordance with the instruction. The reverse transcription kit (Tiangen, Beijing, China) was used to reverse transcribe mRNA to cDNA. A qPCR PreMix kit (Tiangen, Beijing, China) was used to analyze the relative amounts of mRNA with reference to GAPDH. Primer sequences were described in Supplementary Table S1.

### 2.9. Statistical Analysis

All the statistical differences were calculated by the software GraphPad Prism version 8.3.0. All data are presented as mean ± SEM. Statistical significance among multiple groups was analyzed using one-way analysis of variance (ANOVA) or Kruskal–Wallis test. *p*-values less than 0.05 were considered statistically significant.

## 3. Results

### 3.1. Hepatoprotective Effects of Ala-Gln on MCD-Induced NASH Mice

**Figure 1:**
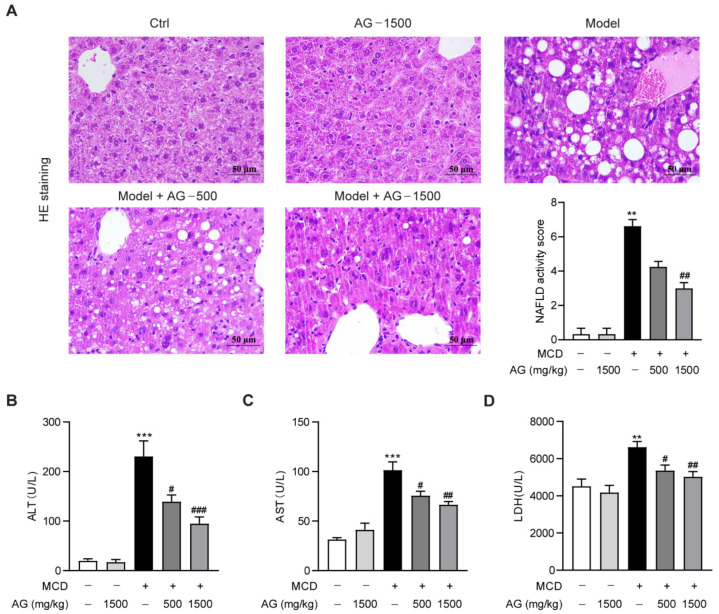
Ala-Gln alleviated steatohepatitis in MCD-fed mice. (**A**) Hematoxylin and eosin (HE) staining (×400) images in liver tissues; quantification of NAFLD activity score. (**B**) Plasma ALT levels. (**C**) Plasma AST levels. (**D**) Plasma LDH levels. Data are expressed as the mean ± SEM. ** *p* < 0.01, *** *p* < 0.001, compared to the Ctrl group; # *p* < 0.05, ## *p* < 0.01, ### *p* < 0.001, compared to the Model group.

To investigate the therapeutic effect of Ala-Gln in NASH, different doses of Ala-Gln (500 or 1500 mg/kg) were administered to MCD-diet-induced NASH mice. First, we examined the effect of Ala-Gln on liver histopathological changes in MCD-diet-fed mice by HE staining. Compared to those of the normal-diet-fed mice, HE staining showed that the hepatic lobules in MCD-diet-fed mice were structurally disturbed, together with marked vacuolar degeneration and inflammatory cells infiltration. In contrast, both doses of Ala-Gln treatment showed significant improvement in liver pathological morphology (Figure 1A). ALT, AST, and LDH can be used as important indexes for liver function evaluation; our data showed that the MCD diet induced a significant up-regulation in plasma ALT, AST, and LDH levels in mice, while Ala-Gln administration dose-dependently reduced these plasma biochemical indexes, which further confirmed that Ala-Gln obviously ameliorated steatohepatitis in mice fed with an MCD diet (Figure 1B–D).

### 3.2. Ala-Gln Ameliorates MCD-Induced Hepatic Lipid Accumulation by Regulating CD36 and FXR Expression

**Figure 2:**
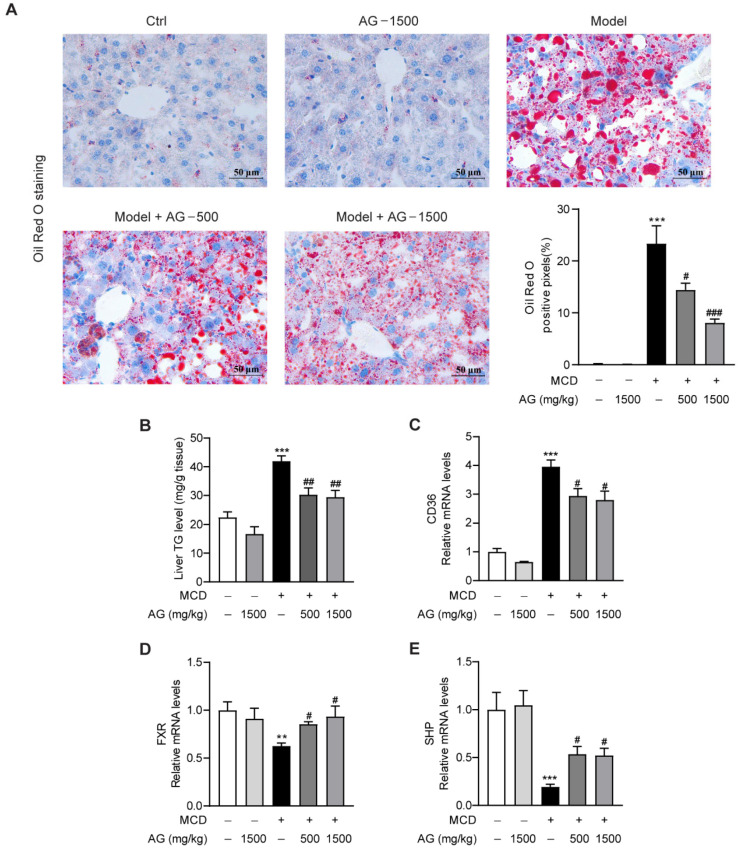
Effects of Ala-Gln on lipid metabolism in MCD-fed mice. (**A**) Oil Red O staining (×400) images in liver tissues; quantification of Oil-Red-O-positive area. (**B**) Liver TG level, (**C**) CD36, (**D**) FXR, and (**E**) SHP mRNA expression in liver. Data are expressed as the mean ± SEM. ** *p* < 0.01, *** *p* < 0.001, compared to the Ctrl group; # *p* < 0.05, ## *p* < 0.01, ### *p* < 0.001, compared to the Model group.

To confirm the regulatory effect of Ala-Gln on hepatic steatosis in MCD-fed mice, we assessed the size and distribution of lipid droplets in the liver using ORO staining. As illustrated, abundant lipid accumulation was observed in the livers of MCD-fed mice. However, Ala-Gln dramatically decreased hepatic fat deposition, while higher doses showed a more pronounced improvement than lower doses, which tended to be more normal (Figure 2A). Meanwhile, the hepatic TG level, which was markedly elevated in NASH model group, showed a dose-dependent decline after Ala-Gln treatment (Figure 2B). To explore the molecular mechanism by which Ala-Gln affects lipid deposition, we analyzed the mRNA levels of genes associated with lipid metabolism. From the data, we observed that Ala-Gln greatly suppressed the mRNA expression level of the fatty acid translocase CD36 (Figure 2C). Furthermore, Ala-Gln also markedly up-regulated the mRNA expression of FXR and SHP (Figure 2D,E). These results demonstrated that Ala-Gln can reduce liver TGs through modulating CD36 and FXR expression.

### 3.3. Ala-Gln Protects against Oxidative Stress in MCD-Fed Mice

**Figure 3:**
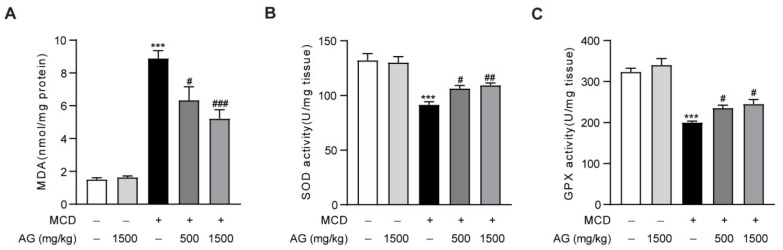
Ala-Gln alleviates hepatic oxidative stress in NASH mice. (**A**) Hepatic MDA levels. (**B**) Hepatic SOD activities. (**C**) Hepatic GPX activities. Data are expressed as the mean ± SEM. *** *p* < 0.001, compared to the Ctrl group; # *p* < 0.05, ## *p* < 0.01, ### *p* < 0.001, compared to the Model group.

Oxidative stress is the main pathological mechanism of NASH and plays an essential role in its onset and progression [19]. To determine the influence of Ala-Gln on oxidative stress in MCD-treated mice, we examined hepatic MDA levels, which is a naturally occurring oxidation product and one of the most commonly used biomarkers of oxidative stress in liver disease [20]. The data showed that MCD-diet feeding in mice induced a significant upregulation of hepatic MDA levels compared to normal-diet feeding in mice, while Ala-Gln treatment significantly reduced this increase of MDA levels in the liver (Figure 3A). Antioxidant enzymes, including SOD and GPX, are extremely vital antioxidant defense systems that defend against reactive oxygen species in the body [21]. In our research, MCD induction led to a down-regulation in hepatic SOD and GPX activities in mice, while Ala-Gln treatment notably reversed these changes and exhibited a concentration-dependent trend (Figure 3B,C). These experiments demonstrated that Ala-Gln treatment significantly ameliorated MCD-induced hepatic oxidative stress characterized in terms of decreased MDA content and elevated SOD and GPX activities.

### 3.4. Ala-Gln Inhibits MCD-Induced Liver Inflammation in Mice

**Figure 4:**
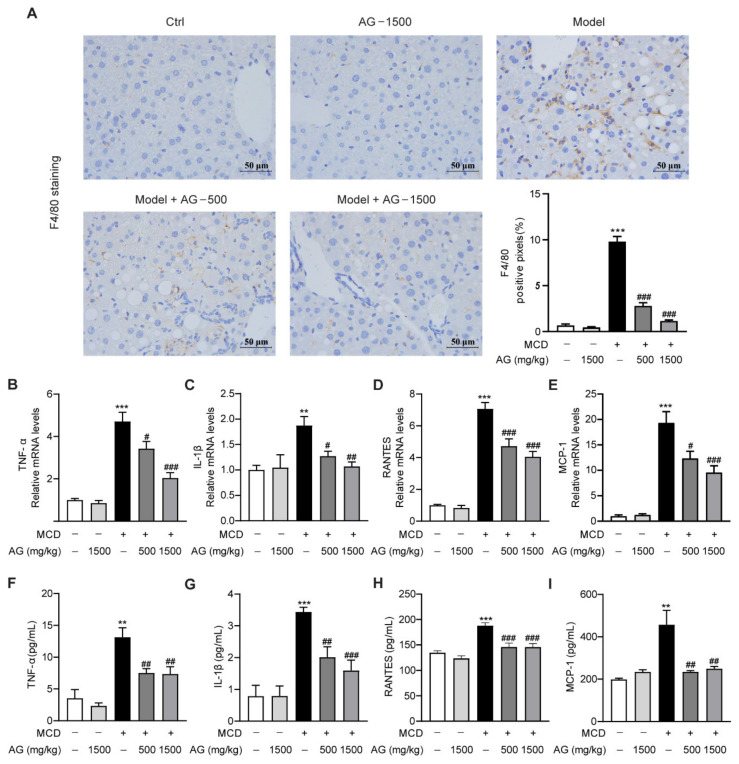
Effects of Ala-Gln on inflammatory response in the liver of MCD-fed mice. (**A**) Representative images for F4/80 staining (×400) in the liver section and quantitative results. (**B**–**E**) The mRNA levels of TNF-α, IL-1β, RANTES, and MCP-1 in liver. (**F**–**I**) The protein levels of TNF-α, IL-1β, RANTES, and MCP-1 in plasma. Data are expressed as the mean ± SEM. ** *p* < 0.01, *** *p* < 0.001, compared to the Ctrl group; # *p* < 0.05, ## *p* < 0.01, ### *p* < 0.001, compared to the Model group.

Inflammatory response has been reported to be a major contributor to NASH progression [22]. To validate whether Ala-Gln can attenuate hepatic macrophages accumulation in MCD-induced NASH mice, IHC staining was performed to examine the expression and distribution of F4/80, which is a surface biomarker of activated macrophages [23]. As indicated in Figure 4A, the MCD diet induced abundant F4/80 distribution in liver tissues, while Ala-Gln dose-dependently decreased hepatic F4/80 expression. Next, real-time qPCR assay revealed that MCD feeding also significantly up-regulated hepatic mRNA levels of pro-inflammatory cytokines and chemokines, comprising TNF-α, IL-1β, RANTES, and MCP-1, while Ala-Gln dramatically attenuated the production of these inflammatory mediators (Figure 4B–E). Similarly, the Elisa assay showed that the elevated expression of TNF-α, IL-1β, RANTES, and MCP-1 in the plasma of MCD-treated mice was also greatly reduced due to Ala-Gln treatment in a concentration-dependent pattern (Figure 4F–I). The above results indicated that Ala-Gln treatment could effectively suppress the MCD-induced hepatic inflammatory response.

### 3.5. Ala-Gln Suppresses MCD-Induced Liver Fibrosis

**Figure 5:**
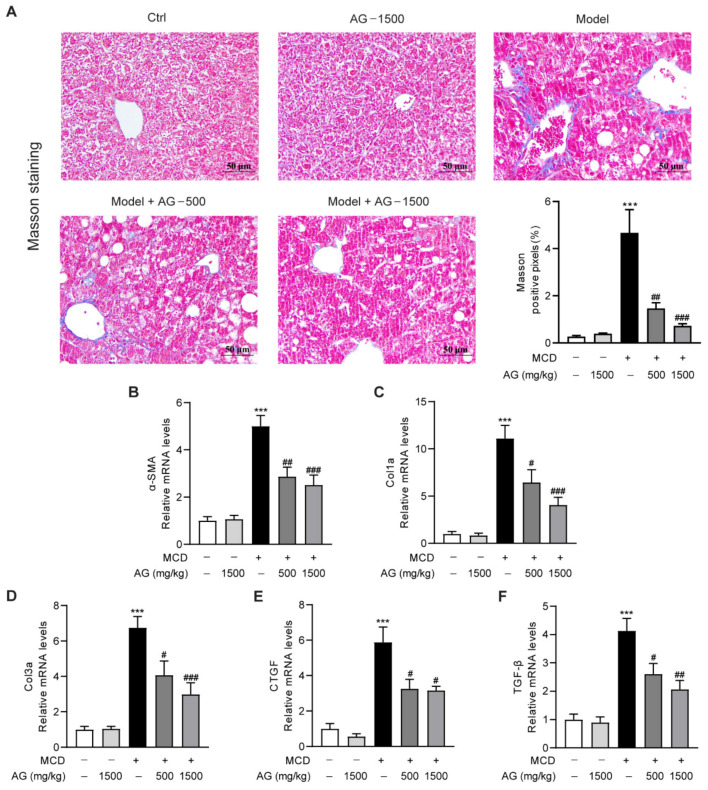
The anti-fibrosis activity of Ala-Gln in MCD-fed mice. (**A**) Representative Masson’s trichrome staining images (×400) in liver. (**B**–**F**) The mRNA levels of α-SMA, Col1a, Col3a, CTGF, and TGF-β in liver. Data are expressed as the mean ± SEM. *** *p* < 0.001, compared to the Ctrl group; # *p* < 0.05, ## *p* < 0.01, ### *p* < 0.001, compared to the Model group.

NASH is typically characterized by progressive destruction and regeneration of hepatocytes, with the development of fibrosis being the most common phenomenon [24]. To explore the modulatory role of Ala-Gln treatment on MCD-triggered hepatic fibrosis in mice, we first analyzed the distribution of collagen fibers in liver by Masson’s trichrome staining. Large collagen expression was found to be visible in MCD-treated mice in comparison with normal-diet-fed mice, whereas the area of positive collagen staining was significantly reduced in Ala-Gln-treated mice (Figure 5A). Meanwhile, we determined the expression of liver profibrogenic genes, comprising α-SMA, Col1a, Col3a, CTGF, and TGF-β. The data suggested that the mRNA levels of these profibrogenic genes were greatly increased after MCD-diet feeding, while Ala-Gln treatment markedly reduced the expression of these genes compared with the MCD group, and all of them displayed a concentration-dependent trend (Figure 5B–F). The above results revealed that Ala-Gln has an anti-fibrosis property in the liver in MCD-induced NASH mice.

**Figure 6:**
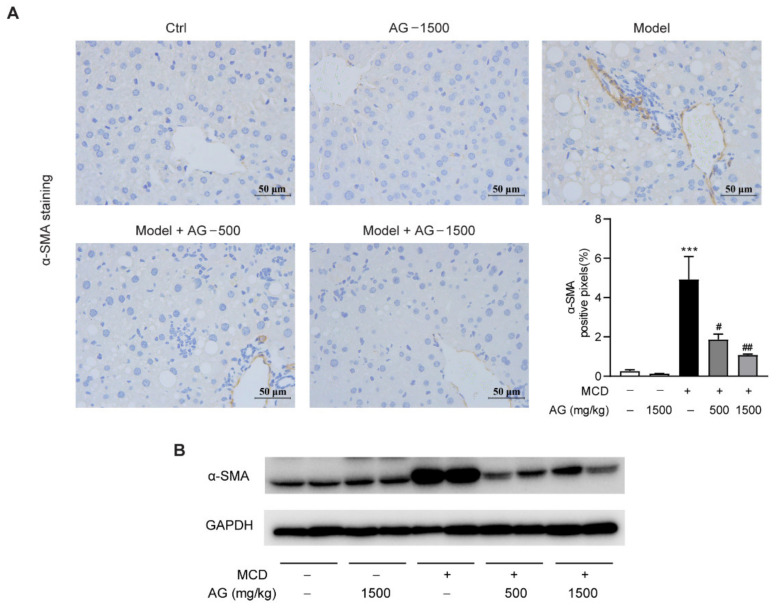
Ala-Gln suppressed hepatic stellate cells activation in MCD-fed mice. (**A**) Representative images for α-SMA staining (×400) in the liver section and quantitative results. (**B**) Hepatic α-SMA protein levels. Data are expressed as the mean ± SEM. *** *p* < 0.001, compared to the Ctrl group; # *p* < 0.05, ## *p* < 0.01, compared to the Model group.

Among above profibrogenic genes, α-SMA is a marker of hepatic stellate cells activation, which contributes to liver fibrosis progression. From the results of IHC staining and Western blotting, we could observe that α-SMA protein expression was significantly elevated in MCD-diet-fed mice in comparison with the mice fed a normal diet, while Ala-Gln treatment significantly attenuated these elevations (Figure 6). These results demonstrated that the anti-fibrosis effect of Ala-Gln may be mainly due to the inhibition of hepatic cells activation.

## 4. Discussion

Herein, we demonstrated a protective effect of different doses of Ala-Gln treatment on NASH. The MCD-diet-feeding model is a model that frequently used to investigate the mechanisms of NASH and to screen pharmacological therapies against NASH, as it can mimic various clinical features of human NASH [25]. By using an MCD-diet-induced rodent NASH model, this study revealed that Ala-Gln treatment could dose-dependently ameliorate hepatic histological steatosis, hepatocytes injury, oxidative stress, inflammation, and fibrosis. Mechanically, Ala-Gln inhibited hepatic oxidative stress through up-regulating antioxidant enzymes including SOD and GPX; suppressed lipid accumulation in liver by regulate the gene expression of CD36, FXR, and SHP; suppressed inflammatory responses via reducing intrahepatic macrophage accumulation and production of proinflammatory factors; and alleviated fibrosis via suppressing the mRNA level of profibrotic genes and inhibiting hepatic stellate cells (HSC) activation.

The pathogenesis of NASH is closely related to lipid accumulation in liver [26]. From HE staining, TG assay, and ORO staining results, it was indicated that Ala-Gln treatment significantly reduced hepatic lipid accumulation. Free fatty acids could enter into cells with the aid of several transporters, including CD36, plasma membrane fatty acid binding protein (FABPpm), and fatty acid transport proteins (FATPs). Among above fatty acid transporters, CD36 is the best characterized, which could promote fatty acid uptake in hepatocytes and thus enhance hepatic lipid accumulation in the progression of NAFLD [27,28]. High hepatic CD36 expression has been observed in NAFLD rodents and patients [29]. In agreement with previous reports, the present data demonstrated that the CD36 expression is higher in MCD-diet-fed mice, while Ala-Gln administration obviously down-regulated the hepatic expression of CD36. Therefore, the down-regulation of CD36 may be an important mechanism contributing to the effect of Ala-Gln to attenuate hepatic steatosis. The nuclear receptor FXR is highly expressed in the liver and plays a key role in the regulation of lipid metabolism [30]. Accumulating evidence suggested that FXR can be an effective target to alleviate the pathogenesis of various liver diseases, including primary biliary cholangitis, nonalcoholic fatty liver disease, and liver fibrosis [31]. Small heterodimer partner (SHP) is the most widely studied target gene of FXR [32]. Our present data showed that Ala-Gln greatly increased hepatic expression of FXR and SHP, suggesting that Ala-Gln can activate FXR signaling pathway in an MCD-diet-induced NASH model. Lipid accumulation can lead to hepatocytes injury [33]. Our data revealed that Ala-Gln not only significantly reduced lipid accumulation in the liver but also obviously decreased ALT, AST, and LDH levels in plasma, indicating the hepatoprotective role of Ala-Gln against hepatocytes injury in MCD-fed mice.

During the development of NASH, the excessive lipid accumulation could cause the dysfunction of mitochondrial function and then exacerbate hepatic oxidative stress, which in turn promotes the aggravation of NASH [34]. Recently, anti-oxidation has been considered as an effective therapy for the prevention and treatment of NASH [35]. Elevated MDA levels and decreased antioxidant enzyme activities in liver can be observed in NASH mice model induced by MCD feeding, as evident in this research [36]. Our previous study and some other reports have found that Ala-Gln plays a key role against oxidative stress in various liver diseases [15,16,37]. Interestingly, Ala-Gln treatment also ameliorated the hepatic oxidative stress in MCD-diet-fed mice in our present study by enhancing antioxidant activities of SOD and GPX. These results demonstrated that the beneficial effects of Ala-Gln on NASH can be partly attributed to its anti-oxidative functions, which were also revealed in previous reports.

Inflammation is recognized as an important mediator contributing to the progression of NASH [26]. Accumulating evidence has indicated that the inflammatory responses that occur in the liver could aggravate the oxidative stress and hepatocytes injury, resulting in more serious pathogenesis of steatohepatitis [38,39]. Most of the pro-inflammatory cytokines in the liver were resourced from intrahepatic macrophages [40]. The number of macrophages in liver was associated with the severity of steatohepatitis in clinical NASH patients [41]. Abundant macrophages accumulation in liver in MCD-diet-fed mice was observed in our present study, while Ala-Gln treatment significantly reversed the increased accumulation of macrophages. It has been suggested that Ala-Gln exhibited anti-inflammatory potential against brain ischemia–reperfusion injury in gerbils [42]. Another study revealed that Ala-Gln reduced LPS-induced inflammation in bovine jejunum epithelial cells [43]. Moreover, our previous study also suggested that LPS-induced hepatic inflammation can be significantly suppressed in mice [16]. Here, we provide evidence that the inhibition of inflammation is associated with the protective effect of Ala-Gln in MCD diet-induced NASH mice.

Another intriguing finding of this study was that Ala-Gln could markedly ameliorate fibrogenesis in liver. Growing evidence has demonstrated that persistent hepatic inflammation could lead to the occurrence of hepatic fibrosis, which is a wound-healing response characterized by collagen deposition in liver [44]. In line with previous reports, the results in the present study showed that MCD feeding led to liver structural disorder and collagen accumulation, accompanied by the elevation of profibrogenic genes including α-SMA, Col1a, Col3a, CTGF, and TGF-β1 [45,46]. On the contrary, Ala-Gln treatment significantly suppressed MCD-diet-induced hepatic fibrosis progression. It was widely reported that hepatic fibrosis was mainly initiated and accelerated by HSC activation [47,48]. The profibrogenic factor TGF-β1 secreted from intrahepatic macrophages could induce HSC transition into myofibroblasts that express α-SMA [49]. In this research, the determination of α-SMA protein level in liver by IHC and WB analysis suggests that MCD diet feeding can promote HSC activation, whereas Ala-Gln greatly suppressed both the gene and protein level of α-SMA in the liver in MCD-diet-fed mice. It can be suggested that the improvement of liver fibrosis after Ala-Gln treatment might be partly due to the inhibition of HSC activation in liver.

## 5. Conclusions

In summary, our present study provided evidence that Ala-Gln is effective for treating NASH in a dose-dependent pattern; the proposed mechanism might be that it could effectively regulate oxidative stress and inflammation. The results support the possibility that Ala-Gln may diminish oxidative stress by enhancing SOD and GPX activities, reducing lipid accumulation via regulating the expression of CD36 and FXR, alleviating inflammation by decreasing the number of activated macrophages and proinflammatory mediators, and suppressing the progression of liver fibrosis through inhibiting hepatic stellate cells activation. Together, these findings demonstrated that Ala-Gln treatment can be considered as a potential strategy in the management of NASH.

## Data Availability

All data generated for this research are included in the article/Supplementary Material.

## Supplementary Materials

The following supporting information can be downloaded at: https://www.mdpi.com/article/10.3390/nu14183796/s1, Table S1: the list of PCR primers used in this study.
