# Supplementary material for: Alanyl-Glutamine Protects Mice against Methionine- and Choline-Deficient-Diet-Induced Steatohepatitis and Fibrosis by Modulating Oxidative Stress and Inflammation
Source: Nutrients. 2022 Sep 15;14(18):3796. doi: 10.3390/nu14183796 (PMC9503574; doi:10.3390/nu14183796)
Supplement: Supplementary file 1 [file nutrients-14-03796-s001.zip › nutrients-1904234-supplementary.pdf]

Table S1. Sequence of primers.

Genes	Forward primer	Reverse primer
TNF-α	caggcggtgcctatgtctc	cgatcaccccgaagttcagtag
IL-1β	gaaatgccaccttttgacagtg	tggatgctctcatcaggacag
MCP-1	ttaaaaacctggatcggaaccaa	gcattagcttcagatttacgggt
RANTES	gctgctttgcctacctctcc	tcgagtgacaaacacgactgc
Col1a	acggctgcacgagtcacac	ggcaggcgggaggtctt
Col3a	gttctagaggatggctgtactaaacaca	ttgccttgcgtgtttgatattc
α-SMA	caggcatggatggcatcaatcac	actctagctgtgaagtcagtgtcg
CTGF	gggcctcttctgcgatttc	atccaggcaagtgcattggta
TGF-β	tgacgtcactggagttgtacgg	ggttcatgtcatggatggtgc
CD36	atgggctgtgatcggaactg	gtcttcccaataagcatgtctcc
FXR	ggcagaatctggatttggaatcg	gcccaggttggaatagtaagacg
SHP	tgggtcccaaggagtatgc	gctccaagacttcacacagtg
GAPDH	cggttccgatgccctgaggctctt	cgtcacacttcatgatggaattga
